# Diagnostic Value of the Impairment of Olfaction in Parkinson's Disease

**DOI:** 10.1371/journal.pone.0064735

**Published:** 2013-05-16

**Authors:** Swaantje Casjens, Angelika Eckert, Dirk Woitalla, Gisa Ellrichmann, Michael Turewicz, Christian Stephan, Martin Eisenacher, Caroline May, Helmut E. Meyer, Thomas Brüning, Beate Pesch

**Affiliations:** 1 Institute for Prevention and Occupational Medicine of the German Social Accident Insurance, Institute of the Ruhr-Universität Bochum (IPA), Bochum, Germany; 2 Neurological Clinic, St. Josef-Hospital, Ruhr-Universität Bochum, Bochum, Germany; 3 Medizinisches Proteom-Center, Ruhr-Universität Bochum, Bochum, Germany; Technical University of Dresden Medical School, Germany

## Abstract

**Background:**

Olfactory impairment is increasingly recognized as an early symptom in the development of Parkinson's disease. Testing olfactory function is a non-invasive method but can be time-consuming which restricts its application in clinical settings and epidemiological studies. Here, we investigate odor identification as a supportive diagnostic tool for Parkinson's disease and estimate the performance of odor subsets to allow a more rapid testing of olfactory impairment.

**Methodology/Principal Findings:**

Odor identification was assessed with 16 Sniffin' sticks in 148 Parkinson patients and 148 healthy controls. Risks of olfactory impairment were estimated with proportional odds models. Random forests were applied to classify Parkinson and non-Parkinson patients. Parkinson patients were rarely normosmic (identification of more than 12 odors; 16.8%) and identified on average seven odors whereas the reference group identified 12 odors and showed a higher prevalence of normosmy (31.1%). Parkinson patients with rigidity dominance had a twofold greater prevalence of olfactory impairment. Disease severity was associated with impairment of odor identification (per score point of the Hoehn and Yahr rating OR 1.87, 95% CI 1.26–2.77). Age-related impairment of olfaction showed a steeper gradient in Parkinson patients. *Coffee*, *peppermint*, and *anise* showed the largest difference in odor identification between Parkinson patients and controls. Random forests estimated a misclassification rate of 22.4% when comparing Parkinson patients with healthy controls using all 16 odors. A similar rate (23.8%) was observed when only the three aforementioned odors were applied.

**Conclusions/Significance:**

Our findings indicate that testing odor identification can be a supportive diagnostic tool for Parkinson's disease. The application of only three odors performed well in discriminating Parkinson patients from controls, which can facilitate a wider application of this method as a point-of-care test.

## Introduction

Parkinson's disease (PD) is a neurodegenerative disorder characterized by movement disorders, such as tremor at rest, bradykinesia, and rigidity, which are mainly due to nigrostriatal dopamine deficiency that can be alleviated by Levodopa [1]. Although degeneration of the substantia nigra pars compacta is considered a neuropathologic hallmark of PD, the neurodegenerative process also includes extranigral structures, such as the olfactory bulb resulting in an impairment of olfaction [2]. More than 90% of PD patients are diagnosed with olfactory impairment. The high prevalence and early occurrence of the olfactory impairment suggest that the test for olfactory dysfunction can be a supportive diagnostic tool for PD [3], [4]. Furthermore, the development of symptoms like tremor or rigidity indicates that PD is a heterogeneous disease with progress in severity. Olfactory impairment may accompany disease progression [5] and vary by subtype based on specific clinical criteria [6].

An optimal diagnostic test with widespread application should be robust and easy to perform. The investigation of odor impairment is such a non-invasive tool that can be applied in research and clinical settings in addition to costly diagnostic methods. A defined set of odorants is consecutively presented to the participant in a multiple forced choice format. Most studies on olfactory impairment in PD, from both the United States and Europe, have focused on odor identification using either the University of Pennsylvania Smell Identification Test (UPSIT) [7] or a test battery with Sniffin' sticks [8], which showed a good agreement with UPSIT [9].

The application of point-of-care tests should not be time consuming to ensure their wide application. Odor identification requires several minutes per odor in order to reduce carry-over effects between the provided odors. Various efforts have been made to estimate the performance of individual odors in the assessment of olfactory impairment for a quicker smell test [10]–[12]. Candidate odors should achieve a similar good discrimination of PD patients compared to the full sets of olfactory probes.

We used Sniffin' sticks to assess odor identification in both PD patients and controls. The present study explores the association of olfactory impairment with disease severity and with the subtype of PD. The performance of single and combined odors to discriminate PD from participants without PD is assessed in comparison to the full set of odors to establish a brief olfaction test.

## Materials and Methods

### Ethics Statement

The study was approved by an independent ethic committee of the Ruhr-Universität Bochum (amendment of AZ3184-08) and conducted according to the principles expressed in the Declaration of Helsinki. All participants were given full information regarding the study, signed to indicate their consent to take part, and could withdraw at any time. Participation, withdrawal or not, had no effect on the health care or other services provided. All comments and information were kept confidential, and patient identifying information was not recorded on the questionnaires, but kept separately from their consent forms.

### Study groups

ParkCHIP was a cross-sectional study in 148 PD patients and 148 controls. The control group was unrelated to PD patients and frequency-matched to cases by gender and age. All controls were free of neurodegenerative diseases. This analysis was conducted in 296 subjects with complete information on odor identification assessed with 16 Sniffin' sticks from January 2010 to September 2011. Subjects who suffered loss of olfaction after surgery, basilar skull fracture, or head trauma were non-eligible for this analysis. Furthermore, subjects with severe cognitive impairment, drug addiction, HIV positive status, or insufficient German language skills were not enrolled for this study. A questionnaire was applied to assess socio-demographic information, medications, and other data. A physician enrolled and diagnosed eligible subjects with written informed consent.

PD patients had a validated diagnosis according to the criteria of the UK Parkinson's Disease Society Brain Bank [13]. Patients receiving dopaminergic medication had to respond to this medication as obligate inclusion criteria in order to improve diagnostic accuracy. Patients who were not under medical treatment underwent functional imaging via Single Photon Emission Computed Tomography to support the diagnosis. Disease severity was assessed using the Unified Parkinson's Disease Rating Scale (UPDRS) [14], the Movement Disorder Society-Sponsored Revision of the Unified Parkinson's Disease Rating Scale (MDS-UPDRS) [15], as well as the Hoehn and Yahr scale [16]. Movement disorders were documented using video recording with the permission of the patient. Physical examination by a neurologist (D.W. or G.E.) determined whether the patients were categorized with ‘tremor dominance’ (N = 38), ‘rigidity dominance’ (N = 90), or as ‘others’ (N = 20).

### Testing odor identification

Sniffin' sticks were applied to assess the identification of 16 odors. Odors were presented in felt-tip pens. The individual pens were consecutively placed in front of both nostrils at a distance of approximately 1–2 cm. The participants could identify the odor as a multiple-choice task from a list of four potential answers [8], [17]. Subjects were classified as normosmic if more than 12 odors were identified, hyposmic if 8 to 12 odors were identified, and anosmic if less than 8 odors were identified [18].

### Examination of disability, motor disorders, and cognitive skills

All participants underwent a routine neurological examination to test for signs of neurological disorders. A physician (D.W. or G.E.) assessed tremor at rest, postural tremor, rigidity, hypokinesia, as well as postural instability. The disability index was scored using the Health Assessment Questionnaire (HAQ) [19]. Cognitive skills were scored by the Mini-Mental State Examination (MMSE) and the clock drawing test [20].

### Statistical analysis

Median and inter-quartile range (IQR) were used to describe the distribution of continuous variables. Study groups were compared using the Kruskal-Wallis test for continuous variables and the chi-square test for categorical data. The relative risks of olfactory impairment (normosmia as reference, hyposmia, and anosmia) were estimated with proportional odds models [21] where the proportional odds ratio (OR) for a predictor can be interpreted as a summary of the risk estimates obtained from separate binary logistic regressions using all cut-points of the ordinal outcome. Age was implemented in groups (<45, 45–65, 66–79, ≥80 years) or as binary variable with 65 years as cut-off. The age-dependent risk of the impairment of olfaction was estimated for diseased and non-diseased subjects with regard to a common reference group (non-diseased subjects aged 45–65) and separately within each study group. We adjusted the association between movement disorders and age for cognitive impairment assessed with the clock drawing test. Random forests with 10-fold cross-validation were performed to evaluate the best discriminating odors between the study groups based on the permutation accuracy using the package randomForest [22], [23] in R 2.13 [23], [24]. For each tree of the random forest the misclassification rate with and without permutation of each odor was recorded. The permutation accuracy was then defined as the difference between the two misclassification rates averaged over all trees and normalized by the standard deviation of the differences. High values of the permutation accuracy indicate important variables. Other analyses were performed with SAS/STAT and SAS/IML software, version 9.2 (SAS Institute Inc., Cary, NC).

## Results

### Description of the study groups

Table 1 presents the characteristics of the PD patients in comparison with the controls. PD patients were on average 67 years old (IQR 59–73 years). The age distribution of controls was similar (IQR 56–72 years). Compared to the controls, a higher proportion of the PD patients were well-educated and never smokers (54.1% *vs*. 40.5% in controls). They showed a good performance in cognitive tests, but had a higher degree of disability compared to the control group. No significant differences, according to these characteristics, were found between the two subgroups of PD, showing either tremor or rigidity dominance (supplemental Table S1).

**Table 1:** Characteristics of patients with Parkinson's disease (PD) and controls.

		PD (N = 148)		Controls (N = 148)		PD *vs*. controls
		N	%/Median (IQR^b^)	N	%/Median (IQR)	P value^a^
Age [years]		148	67 (59;73)	148	62 (56;72)	0.089
Gender	Male	78	52.7	81	54.7	0.727
	Female	70	47.3	67	45.3	
Smoking status	Never	80	54.1	60	40.5	0.006
	Former	58	39.2	62	41.9	
	Current	10	6.8	26	17.6	
Education [years]	<10	75	50.7	82	55.4	0.123
	10	25	16.9	33	22.3	
	>10	48	32.4	33	22.3	
Native speaker	Yes	129	87.2	137	92.6	0.123
	No	19	12.8	11	7.4	
MMSE^c^ excluding manual tasks (max = 24)		147	23 (21;23)	144	23 (22;24)	<0.001
Clock drawing test (max = 7)		147	7 (5;7)	141	7 (5;7)	0.471
Disability index of HAQ^d^ (max = 3)		148	0.5 (0;1.4)	143	0 (0;0)	<0.001

a P value of χ^2^ test for categorical variables and of Kruskal-Wallis test for continuous variables; ^b^Inter-quartile range; ^c^Mini-Mental State Examination; ^d^Health Assessment Questionnaire.

### Odor identification in PD patients and controls

Table 2 depicts the impairment of odor identification in PD patients, who identified on average 7 out of 16 odors, compared to a median of 12 in the controls. Every other PD patient (56.8%) was anosmic in contrast to 6.8% in the control group. Ten PD patients (6.8%) were normosmic and were on average 13 years younger than the other PD patients. Although the difference in odor identification was not statistically significant between the two PD subgroups (p = 0.22), 62.2% of the patients with rigidity dominance were anosmic.

**Table 2:** Odor identification in the study groups assessed with 16 Sniffin' sticks.

	Identified odors with Sniffin' sticks (max = 16)			Normosmia (16–13 odors)		Hyposmia (12–8 odors)		Anosmia (7–0 odors)		
Study groups	N	Median (IQR^a^)	P value^b^	N	%	N	%	N	%	P value^c^
Parkinson patients (PD)	148	7 (5;9)	<0.001	10	6.8	54	36.5	84	56.8	<0.001
Controls	148	12 (10;13)		46	31.1	92	62.2	10	6.8	
Tremor dominance (PD)	38	7.5 (5;9)	0.216	2	5.3	17	44.7	19	50.0	0.337
Rigidity dominance (PD)	90	6 (4;9)		6	6.7	28	31.1	56	62.2	

a Inter-quartile range; ^b^P value of Kruskal-Wallis test for PD *vs*. controls and for PD with tremor dominance *vs*. PD with rigidity dominance; ^c^P value of χ^2^ test for PD *vs*. controls and for PD with tremor dominance *vs*. PD with rigidity dominance.

Proportional odds models revealed that age was a strong confounder in both groups and showed a non-linear shape of the impairment of odor identification assessed as anosmia or hyposmia (Table 3). Female gender and current smoking were associated with improved odor identification; however, the association was not significant (data not shown). A lower cognitive performance assessed with the clock drawing test (OR 0.83 (95% confidence interval (CI) 0.71–0.98)) but not with the MMSE (OR 0.96 (95% CI 0.85–1.09)) was significantly related to an impairment of odor identification.

**Table 3:** Estimates of proportional odds ratios (OR) with 95% confidence intervals (CI) of Parkinson's disease and age with joint effects on impairment of olfaction (anosmia or hyposmia) adjusted by the clock drawing test result.

		N	OR	95% CI		OR^a^	95% CI	
Controls	<45 years	7	0.25	0.05	1.38	0.16	0.03	0.96
	45–65 years	71	1	Reference group		1	Reference group	
	66–79 years	56	1.19	0.59	2.40	1.38	0.70	2.73
	≥80 years	7	11.24	2.23	56.76	5.32	0.59	47.73
Parkinson patients	<45 years	5	1.30	0.21	7.95	0.23	0.04	1.29
	45–65 years	60	12.74	5.81	27.94	1	Reference group	
	66–79 years	73	18.00	8.31	38.98	1.23	0.60	2.53
	≥80 years	9	74.01	8.17	670.68	17.49	3.10	98.66

a Proportional odds ratios with 95% CI estimated separately for each study group.

Table 3 presents the joint effects of study group and age on olfactory impairment, adjusted for cognitive performance using the clock drawing test. In addition, we estimated proportional odds of the age-related impairment of odor identification for each study group. PD patients aged 45 to 65 years were 12.74 (95% CI 5.81–27.94) times more likely to show anosmia or hyposmia compared to controls within the same age group, and PD patients aged ≥80 years had an OR of 74.01 (95% CI 8.17–670.68). The corresponding OR for impairment of olfaction was in controls aged ≥80 years 17.49 (95% CI 3.10–98.66) in comparison to controls aged 45 to 65 years. Comparing aged PD patients (≥80 years) with younger PD patients (45–65 years), the relative change in impairment was 5.32 (95% CI 0.59–47.73).

Table 4 shows these joint effects for the subgroups of PD using controls aged <65 years as the reference group. PD patients with rigidity dominance had higher (but not statistically significant) ORs for olfactory impairment than patients with tremor dominance, even at a younger age (<65 years: 12.72, 95% CI 5.67–28.53 *vs*. 6.56, 95% CI 2.12–20.35).

**Table 4:** Estimates of proportional odds ratios (OR) with 95% confidence intervals (CI) of subgroups of Parkinson's disease (PD) and age with joint effects on impairment of olfaction (anosmia and hyposmia) adjusted by the clock drawing test result.

		N	OR	95% CI	
Controls	<65 years	78	1.00	Reference group	
	≥65 years	63	1.60	0.82	3.10
PD with rigidity dominance	<65 years	45	12.72	5.67	28.53
	≥65 years	44	30.28	12.45	73.67
PD with tremor dominance	<65 years	15	6.56	2.12	20.35
	≥65 years	23	18.63	6.73	51.60
Other PD patients	<65 years	5	19.14	2.85	128.49
	≥65 years	15	7.36	2.37	22.88

The relative risk of olfactory impairment increased with disease severity in PD patients (Table 5). These changes were significant for the Hoehn and Yahr rating scale (per score point: OR 1.87; 95% CI 1.26–2.77) and the Clinician Global Impression of Disease Severity (per score point: OR 1.65; 95% CI 1.06–2.57). The results were marginally significant for the Unified Parkinson's Disease Rating Scale (UPDRS) and the Movement Disorder Society-Sponsored Revision of the Unified Parkinson's Disease Rating Scale (MDS-UPDRS). The association with the UPDRS-III motor scale was weaker.

**Table 5:** Estimates of proportional odds ratios (OR) with 95% confidence intervals (CI) of disease severity on impairment of olfaction (anosmia and hyposmia) in Parkinson patients with age adjustment.

	Change per	N	OR	95% CI	
Unified Parkinson's Disease Rating Scale (ON, max. score = 199)	33 score points	145	1.75	0.99	3.09
UPDRS-III motor scale (max. score = 108)	18 score points	148	1.38	0.88	2.16
Movement Disorder Society-Sponsored Revision of the Unified Parkinson's Disease Rating Scale (max. score = 260)	43 score points	145	1.78	0.99	3.21
Hoehn and Yahr rating (max. score = 5)	1 score point	148	1.87	1.26	2.77
Clinician Global Impression of Disease Severity (max. score = 6)	1 score point	115	1.65	1.06	2.57

Table 6 depicts the group differences in identifying individual odors. *Peppermint* and *fish* ranked highest among the odors that were correctly identified by the controls. The odor of *apples* was rarely identified by both groups. Apart from *cinnamon* and *garlic*, PD patients showed a significant impairment in the ability to identify all other single odors when compared to the controls. *Coffee*, *peppermint*, and *anise* showed the largest differences between both groups. PD subgroups similarly identified most odors, including *peppermint* (p value 0.82), but differed in the ability to smell *oranges*.

**Table 6:** Correctly identified odors in the study groups sorted by the difference between controls (HC) and patients with Parkinson's disease (PD).

	Total	%(HC) minus %(PD)	HC	PD	PD *vs*. HC	PD with tremor dominance	PD with rigidity dominance	Tremor *vs*. rigidity dominance
	%	%	%	%	P value^a^	%	%	P value^a^
Coffee	59.0	44.6	77.7	33.1	<0.001	39.5	31.1	0.360
Peppermint	77.9	40.6	93.9	52.7	<0.001	50.0	52.2	0.818
Anise	61.6	39.2	73.7	34.5	<0.001	26.3	37.8	0.212
Banana	68.3	36.5	84.5	48.0	<0.001	52.6	45.6	0.464
Licorice	68.9	35.8	85.1	49.3	<0.001	50.0	45.6	0.645
Fish	80.7	31.1	95.3	64.2	<0.001	71.1	58.9	0.194
Leather	63.4	30.4	78.4	48.0	<0.001	55.3	45.6	0.315
Clove	68.7	29.0	82.4	53.4	<0.001	47.4	52.2	0.616
Rose	78.1	26.3	91.2	64.9	<0.001	68.4	61.1	0.433
Orange	78.5	23.0	88.5	65.5	<0.001	79.0	57.8	0.023
Pineapple	38.7	20.3	46.0	25.7	<0.001	29.0	23.3	0.503
Lemon	34.1	18.9	37.8	18.9	<0.001	21.1	11.1	0.139
Turpentine	42.2	15.5	47.3	31.8	0.006	21.1	33.3	0.165
Apple	19.1	14.9	25.7	10.8	<0.001	0	14.4	0.013
Garlic	74.9	14.2	81.1	66.9	0.006	63.2	66.7	0.703
Cinnamon	45.2	−4.1	40.5	44.6	0.481	55.3	41.1	0.142

a P value of Kruskal-Wallis tests.

The misclassification rate of the full set of odors was 22.4% for PD *vs*. control group. Table 7 shows the misclassification rates of the study groups when using subsets of the Sniffin' sticks. *Peppermint*, *anise*, and *coffee* were the top-3 odors based on the highest variable importance according to random forests, and achieved a similar misclassification rate (23.8%). A 5-odor set was proposed by Mueller and colleagues and consists of *orange*, *leather*, *peppermint*, *rose*, and *fish* [12]. This short test yielded similar rates (24.1%). Hummel's quick olfactory test (q-Sticks) [11] containing *coffee*, *clove*, and *rose* led to a misclassification rate of 27.0%, and non-food odors were associated with a rate of 31.9%. The Sniffin' sticks were less successful in the classification of PD subtypes (data not shown).

**Table 7:** Classification of patients with Parkinson's disease (PD) based on Sniffin' sticks using random forests on all and selected odors with ten-fold cross-validation.

	PD *vs*. controls	
Sniffin' sticks	Misclassification [%]	Top-3 odors
16 odors	22.4	Peppermint, coffee, anise
Data-driven top-3 odors^a^	23.8	Peppermint, coffee, anise
Peppermint	29.6	
Coffee	27.6	
Anise	30.3	
Fish	34.6	
Licorice	32.6	
Lemon	40.1	
Non-food odors (leather, turpentine, rose)	31.9	Leather, rose, turpentine
Q-sticks (coffee, clove, rose)^b^	27.0	Coffee, rose, clove
5-odor set (orange, leather, peppermint, rose, fish)^c^	24.1	Peppermint, fish, rose
3-odor set (cinnamon, licorice, anise)^d^	26.4	Anise, licorice, cinnamon

a Odors with the three highest variable importance measures according to random forest; ^b^Hummel et al. 2010; ^c^Mueller et al. 2006; ^d^Boesveldt et al. 2008.

**Figure 1:**
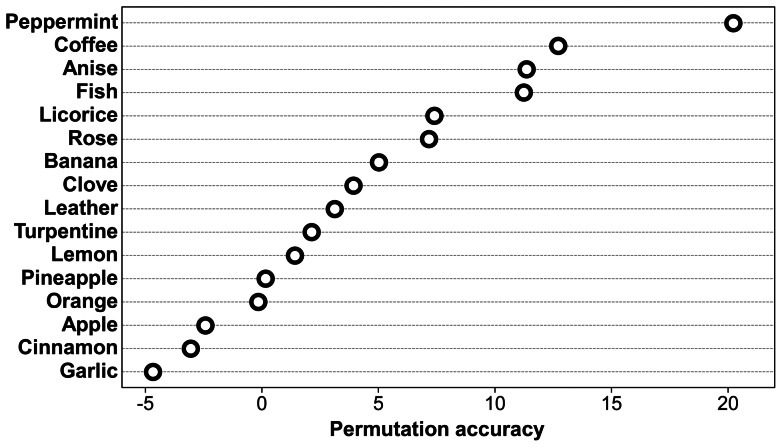
Ranking of the odors with regard to their importance in achieving a good accuracy of classification based on the permutation accuracy of random forests. High values of the permutation accuracy indicate important variables.

Figure 1 shows the ranking of the odors with regard to their importance in achieving a good accuracy of classification based on the permutation accuracy of random forests. *Peppermint* and *coffee* ranked highest, followed by *anise* and *fish*, whereas odors with negative values like *apple, cinnamon and garlic* did not contribute to an accurate classification of the study groups.

We estimated the sensitivity and specificity of the quick 3-odor test with *peppermint*, *anise*, and *coffee* depending on the decision rule for odor impairment. A specificity of 99% and a sensitivity of 28% were achieved for the classification of PD and controls when the decision rule for impairment was to identify none of the three odors correctly. A higher sensitivity of 61% was possible at the expense of specificity (88%) when the decision rule allows the detection of one of the three odors.

## Discussion

PD is the second most common neurodegenerative disease where point-of-care tests for early detection hold great promise in adjunct to more costly investigations for an improvement of the diagnostic accuracy [1]. Screening tools for early detection of any disease require simple and non-invasive methods before symptoms actually become detectable, usually signs of more severe damage. In general, motor symptoms occur late in PD, when the nigostriatal dopaminergic system is degenerated to a larger extent, whereas an impairment of olfaction is considered an early sign of PD [4]. Odor identification can be performed with robust tests. For example, the ParkCHIP study aimed at improving the classification of PD with diagnostic markers. Here, we evaluated the performance of a brief odor-identification test based on Sniffin' sticks as a supportive diagnostic tool. The present analysis confirmed that odor impairment is highly prevalent in PD patients, for example when compared with the prevalence rates estimated in large population surveys in older adults [25]–[27]. Our statistical model demonstrated that subsets of three to five odors have a similar good performance to discriminate PD compared to the full set of 16 sticks. A brief examination allows a wider application of olfaction testing as a quick point-of-care test in clinical settings and large epidemiological studies.

Our results revealed that various brief test versions of odor identification are sufficiently sensitive to detect severe olfactory dysfunction. This raises the question of which odors should be selected for a quick test. Our top-3 set contained food-related odors, whereas non-food odors had a lower performance of classifying PD, which is in line with a large population survey [27]. Our statistical approach revealed *peppermint*, *coffee*, and *anise* as the best-performing 3-odor set for the discrimination of PD patients from all participants without PD. We also found these odors among other sets that have been previously suggested as brief tests. Usually, these sets of odors were not derived from statistical modeling. If several odors perform well like *anise* and *fish*, the statistical approach selects the best ones from the specified dataset of a defined study population. The application of the same statistical model to other study populations may reveal slightly different top-3 sets. For example, *coffee* together with *rose* and *clove* have been proposed as another quick test [11]. This odor set showed only a slightly different misclassification rate due to the lower identification rate of *anise*, compared to *rose* and *clove* among our PD patients. *Anise* is among the top-3 odors of another study together with *cinnamon* and *licorice* [10]. This set led to a slightly higher misclassification rate because *cinnamon* was identified by 44.6% of the PD patients. *Peppermint* was part of a 5-odor set together with *fish*, *rose*, *orange*, and *leather* [12], [27]. An odor can only perform well in a classification algorithm if a high fraction of the controls can identify this odor correctly in combination with a low proportion of PD patients that is still able to identify this odor. The strong odors of *peppermint* and *fish* were identified in about 90% of the control patients, which confirms an investigation in healthy Dutch subjects [28]. In summary, a quick 3-odor test detects a severe olfactory dysfunction rather than a selective olfactory disability in PD patients.

Sensitivity and specificity are popular measures of the performance of tests as a diagnostic classifier. A high specificity is important for screening tools to avoid invasive or cost-intensive diagnostic workups when subjects are tested positive. When applying a strong decision rule, i.e. none of the three best-classifying odors was detected, we achieved very good specificity of 99% at a sensitivity of nearly 30%. This supports that olfaction testing can serve as a good diagnostic adjunct to other examinations.

Overall, the Sniffin' sticks successfully discriminated PD patients from non-diseased subjects. Despite a generally good classification of PD patients from controls based on their impairment of olfaction, ten out of 148 PD cases were tested normosmic, of all whom were on average younger. Age was a strong confounder, which was already shown in various population surveys [25], [29]. The shape of the age-related impairment showed a non-linear trend. The estimate of the age-related impairment of odor identification increased steeply up to a very high OR in PD patients compared with controls aged 45–65 years. However, the relative change in loss of odor identification was less strong when compared with patients aged 45–65 years. These younger patients had already a significant impairment of their olfaction compared to the controls of a similar age.

We further found an association between disease severity assessed with various scales and olfactory impairment. This observation is in line with a report by Morley and coworkers [30], but contradicts the findings of others where such a relationship could not be detected [3], [31]. The characteristics of the study population, the size of the study, the applied test and scales, and an adjustment for observer or study center could influence whether such an association can be captured. In ParkCHIP, there were only two raters from the same hospital, and all patients fulfilled the criteria for a diagnosis of PD. This association was somewhat stronger for the Hoehn and Yahr scale and the Clinician Global Impression of Disease Severity scale. Both are brief scales in contrast to UPDRS or related scales. Larger scales include unspecific questions, for example on depression, which may attenuate the score. Also, Tissingh and colleagues observed a significant impact of the Hoehn and Yahr stage on the ability to discriminate odors, but not for the UPDRS motor scale [5].

Furthermore, we observed a slightly higher risk of olfactory impairment in PD patients with rigidity dominance when using the full set of Sniffin' sticks that was not statistically significant. This is in line with Stern et al. and Iijima et al. [6], [32] but in contrast to others [3], [33]. Studies with PD patients are often not very large in size; therefore when analyzing subgroups, the statistical power is limited to ensure that a small difference in olfaction is not a spurious finding.

Several other factors may also influence olfactory impairment [34]. In line with other studies, we observed an association between impairment of olfaction and cognitive function [35], [36]. Again, the choice of a sensitive test plays a role. Here, the clock drawing test but not the MMSE showed a significant association. We found some indication for lower risks of olfactory impairment in women and current smokers, supporting results from previous large population-based studies [29], [37].

## Conclusions

Our results provide further support of an association between olfactory impairment as a non-motor manifestation and severity of PD. Brief odor identification tests perform well in discriminating PD and can likely be used as a point-of-care test in clinical settings, together with other examinations in PD diagnostics or in epidemiological studies exploring risk factors for neurodegeneration. A brief test of odor identification is a non-invasive, robust and cost efficient method, which might increase the acceptance among patients and healthy subjects.

## Supporting Information

Table S1 **Characteristics of patients with Parkinson's disease and dominance of tremor or rigidity.**
(DOC)
