# Supplementary material for: Diagnostic Value of the Impairment of Olfaction in Parkinson's Disease
Source: PLoS One. 2013 May 16;8(5):e64735. doi: 10.1371/journal.pone.0064735 (PMC3655992; doi:10.1371/journal.pone.0064735)
Supplement: Table S1 — Characteristics of patients with Parkinson's disease and dominance of tremor or rigidity. (DOC) [file pone.0064735.s001.doc]

**Table S1.** Characteristics of patients with Parkinson’s disease and dominance of tremor or rigidity.

|  | | Tremor dominance (N=38) | | Rigidity dominance (N=90) | | No dominance (N=20) | |  |
| --- | --- | --- | --- | --- | --- | --- | --- | --- |
|  |  | N | % / Median (IQR^a^) | N | % / Median (IQR) | N | % / Median (IQR) | P value^b^ |
| Age [years] |  | 38 | 68 (61;73) | 90 | 65 (57;72) | 20 | 69 (64;74) | 0.309 |
| Gender | Male | 19 | 50.0 | 50 | 55.6 | 9 | 45 | 0.644 |
|  | Female | 19 | 50.0 | 40 | 44.4 | 11 | 55 |  |
| Smoking status | Never | 22 | 57.9 | 46 | 51.1 | 12 | 60 | 0.897 |
|  | Former | 13 | 34.2 | 38 | 42.2 | 7 | 35 |  |
|  | Current | 3 | 7.9 | 6 | 6.7 | 1 | 5 |  |
| Education [years] | < 10 | 22 | 57.9 | 42 | 46.7 | 11 | 55 | 0.459 |
|  | 10 | 8 | 21.1 | 14 | 15.6 | 3 | 15 |  |
|  | > 10 | 8 | 21.1 | 34 | 37.8 | 6 | 30 |  |
| Native speaker | Yes | 34 | 89.5 | 78 | 86.7 | 17 | 85 | 0.867 |
|  | No | 4 | 10.5 | 12 | 13.3 | 3 | 15 |  |
| MMSE^c^ excluding manual tasks (max=24) |  | 38 | 22.5 (21;23) | 89 | 23 (21;23) | 20 | 22 (20;23) | 0.407 |
| Clock drawing test (max=7) |  | 38 | 7 (5;7) | 89 | 7 (5;7) | 20 | 7 (5.5;7) | 0.871 |
| Disability index of HAQ^d^ (max=3) |  | 38 | 0.4 (0;1.3) | 90 | 0.6 (0.1;1.5) | 20 | 0.3 (0.1;1.1) | 0.464 |

^a^Inter-quartile range; ^b^P value of χ² test for categorical variables and of Kruskal-Wallis test for continuous variables; ^c^Mini-Mental State Examination; ^d^Health Assessment Questionnaire.
